# A synthetic mammalian network to compute population borders based on engineered reciprocal cell-cell communication

**DOI:** 10.1186/s12918-015-0252-1

**Published:** 2015-12-30

**Authors:** Katja Kolar, Hanna M. Wischhusen, Konrad Müller, Maria Karlsson, Wilfried Weber, Matias D. Zurbriggen

**Affiliations:** Faculty of Biology, University of Freiburg, DE-79104 Freiburg, Germany; Present address: DMK GmbH, Head of Quality Assurance, DE-26939 Ovelgönne, Germany; Present address: Novartis Pharma AG, Biologics Process R&D, CH-4002 Basel, Switzerland; Present address: Respiratory, Inflammation and Autoimmunity (RIA) iMED, AstraZeneca, SE-431 83 Mölndal, Sweden; BIOSS Centre for Biological Signalling Studies, University of Freiburg, DE-79104 Freiburg, Germany; Present address: Institute of Synthetic Biology and Cluster of Excellence on Plant Science (CEPLAS), University of Düsseldorf, DE-40225 Düsseldorf, Germany

**Keywords:** Synthetic biology, Intercellular communication, Population borders, Edge-detect

## Abstract

**Background:**

Multicellular organisms depend on the exchange of information between specialized cells. This communication is often difficult to decipher in its native context, but synthetic biology provides tools to engineer well-defined systems that allow the convenient study and manipulation of intercellular communication networks.

**Results:**

Here, we present the first mammalian synthetic network for reciprocal cell-cell communication to compute the border between a sender/receiver and a processing cell population. The two populations communicate via L-tryptophan and interleukin-4 to highlight the population border by the production of a fluorescent protein. The sharpness of that visualized edge can be adjusted by modulating key parameters of the network.

**Conclusions:**

We anticipate that this network will on the one hand be a useful tool to gain deeper insights into the mechanisms of tissue formation in nature and will on the other hand contribute to our ability to engineer artificial tissues.

**Electronic supplementary material:**

The online version of this article (doi:10.1186/s12918-015-0252-1) contains supplementary material, which is available to authorized users.

## Background

Multicellular organisms strongly depend on the communication between specialized cells starting from their development throughout their entire lifespan [1]. This communication between cell populations is often hard to decipher in its native context due to the presence of perturbing factors that are not central to the communication but attenuate it in various ways and because of a crosstalk with other signaling components. To gain deeper insight into such complex processes, synthetic biology provides us with tools to engineer artificial intercellular communication systems with a clearly defined, finite number of well-characterized components [2–5]. Such synthetic systems do not only open up the possibility to study and manipulate the communication between cell populations without any external perturbing influences, but are also important steps towards the engineering of artificial tissues. To date, several intercellular communication systems have been constructed in bacteria and in unicellular eukaryotes [2, 6], but the implementation of such systems in mammalian backgrounds is lagging behind, likely due to their unequally greater complexity. The few existing synthetic sender-receiver systems for mammalian cells use acetaldehyde [6], nitric oxide [7], hepatocyte growth factor [8], L-tryptophan [9] or Delta-Notch-mediated direct cell-cell contact [10] as signals for communication. Notably, one system for two-way communication between mammalian cells has been implemented that is based on L-tryptophan and acetaldehyde as signaling molecules. This system has been applied to coordinate the expression of highly regulated factors required for the maturation of blood vessels, namely vascular endothelial growth factor and angiopoietin-1 [9].

One particular example of intercellular communication is the exchange of information between adjacent cell populations in order to define and detect population borders. The importance of this mechanism is probably best illustrated in cancer cells that lose the ability to detect and react to such borders during unregulated tumor growth and metastasis [11, 12]. In this study, we aimed to engineer a synthetic system that emulates the detection of the border between two distinct populations of cells.

## Results and discussion

**Fig. 1:**
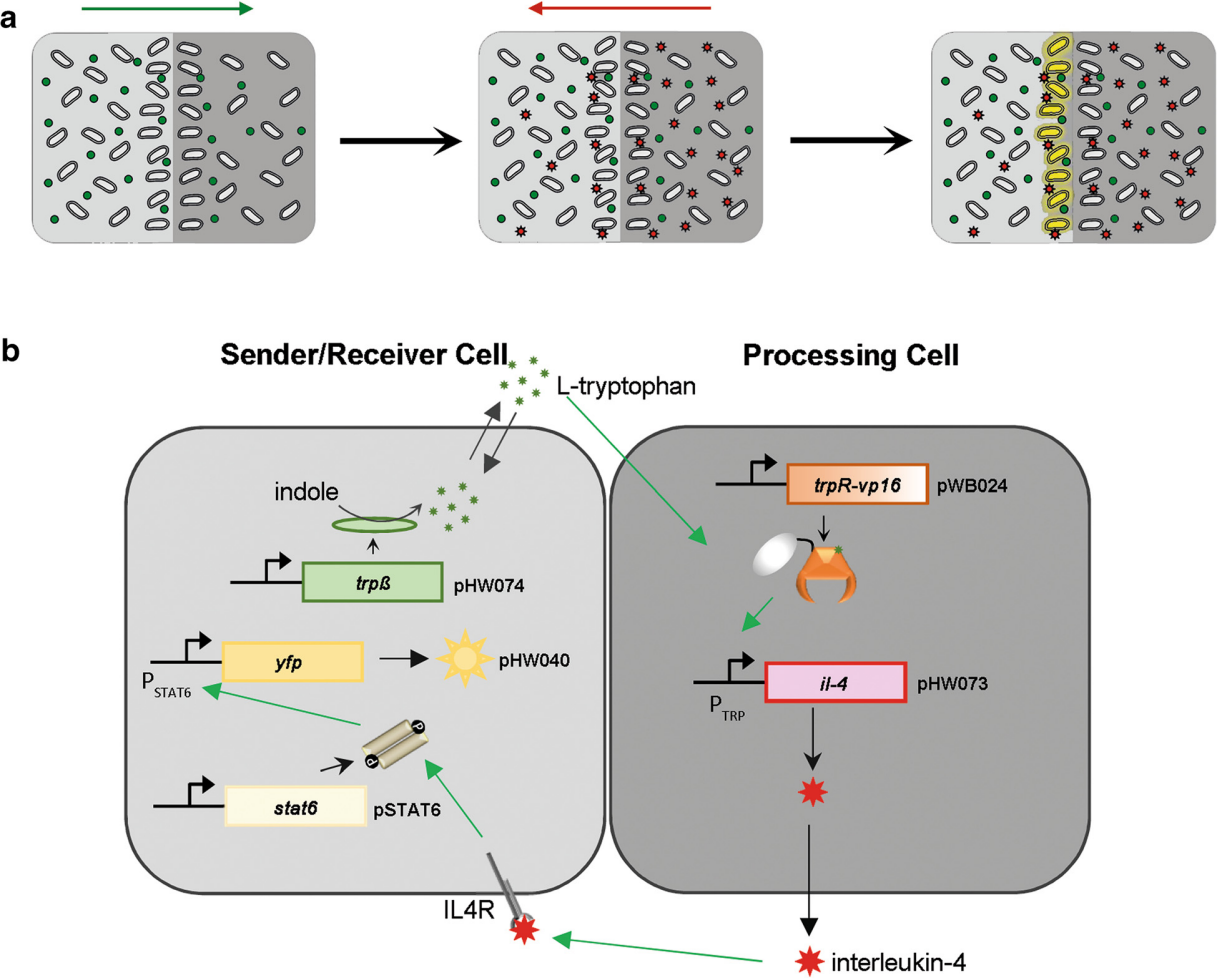
Design of the synthetic network to compute population borders. **a** Mechanistic background. The sender/receiver cell population (light grey) produces a signal (green spheres) that diffuses across the population border into the processing cell population (dark grey). The processing cell population responds by the production of a second signal (red stars) that diffuses back into the sender/receiver population, where it elicits a response from cells at the edge of the two cell populations that are exposed to high enough concentrations of the signaling molecule. **b** Molecular configuration of the synthetic network. The sender/receiver cells produce L-tryptophan from indole via constitutively expressed tryptophan synthase (TrpB). L-tryptophan diffuses into the processing population and is sensed by the chimeric TrpR-VP16 transcription factor that in turn triggers production of interleukin-4 (IL-4). Interleukin-4 diffuses back into the sender/receiver cell population, where it is perceived by the endogenous interleukin-4 receptor (IL4R). The signal is relayed onwards via exogenously expressed STAT6 that finally triggers production of a yellow fluorescent reporter protein

The design of our network is based on two distinct populations of cells that communicate via defined signaling molecules. The sender/receiver cell population produces the first communication signal that diffuses across the population border and is perceived by the processing cell population (Fig. 1a). This cell population responds by the production of the second communication signal that diffuses back into the sender/receiver cell population, which is able to respond to it in a dose-dependent manner, eliciting a maximal response at the population border where the concentration of the signaling molecule surpasses a defined threshold (Fig. 1a). On the molecular level, we used the single available two-way communication system for mammalian cells as the blueprint for our network [9]. This system relies on L-tryptophan and acetaldehyde as signaling molecules. Since acetaldehyde is a gaseous substance, it was not possible to directly apply this system for the computation of population borders, where the spatially-defined diffusion of the signaling molecules is crucial. It has been demonstrated that cells, harboring the type II interleukin-4 receptor but lacking the downstream signaling pathway, can be engineered to respond to interleukin-4 by the expression of human STAT6 [13, 14]. Therefore, we decided to substitute acetaldehyde with interleukin-4 and based our synthetic network for reciprocal cell-cell communication on the production and perception of the signaling molecules L-tryptophan and interleukin-4 (Fig. 1b). In this setup, a sender/receiver cell population converts indole to L-tryptophan via constitutively-expressed tryptophan synthase β from *E. coli* (TrpB) [9]. L-tryptophan diffuses across the population border into the processing cell population, where it is perceived by a fusion protein of the DNA-binding domain of the tryptophan repressor (TrpR) from *Chlamydia trachomatis* and the VP16-transactivation domain from *Herpes simplex* virus [9]. Upon binding of L-tryptophan, the TrpR domain binds to its cognate operator sequence in a response construct and the production of the second signaling molecule, interleukin-4, is initiated via the VP16 domain. Interleukin-4 diffuses back across the population border and is perceived by the sender/receiver cell population via the interleukin-4 receptor, the signal is relayed onwards via STAT6 that finally activates the expression of a gene encoding the fluorescent reporter protein YFP. As the magnitude of YFP gene expression is correlated to the interleukin-4 concentration, the sharpness of the visualized edge can be adjusted by tuning the interleukin-4 levels.

**Fig. 2:**
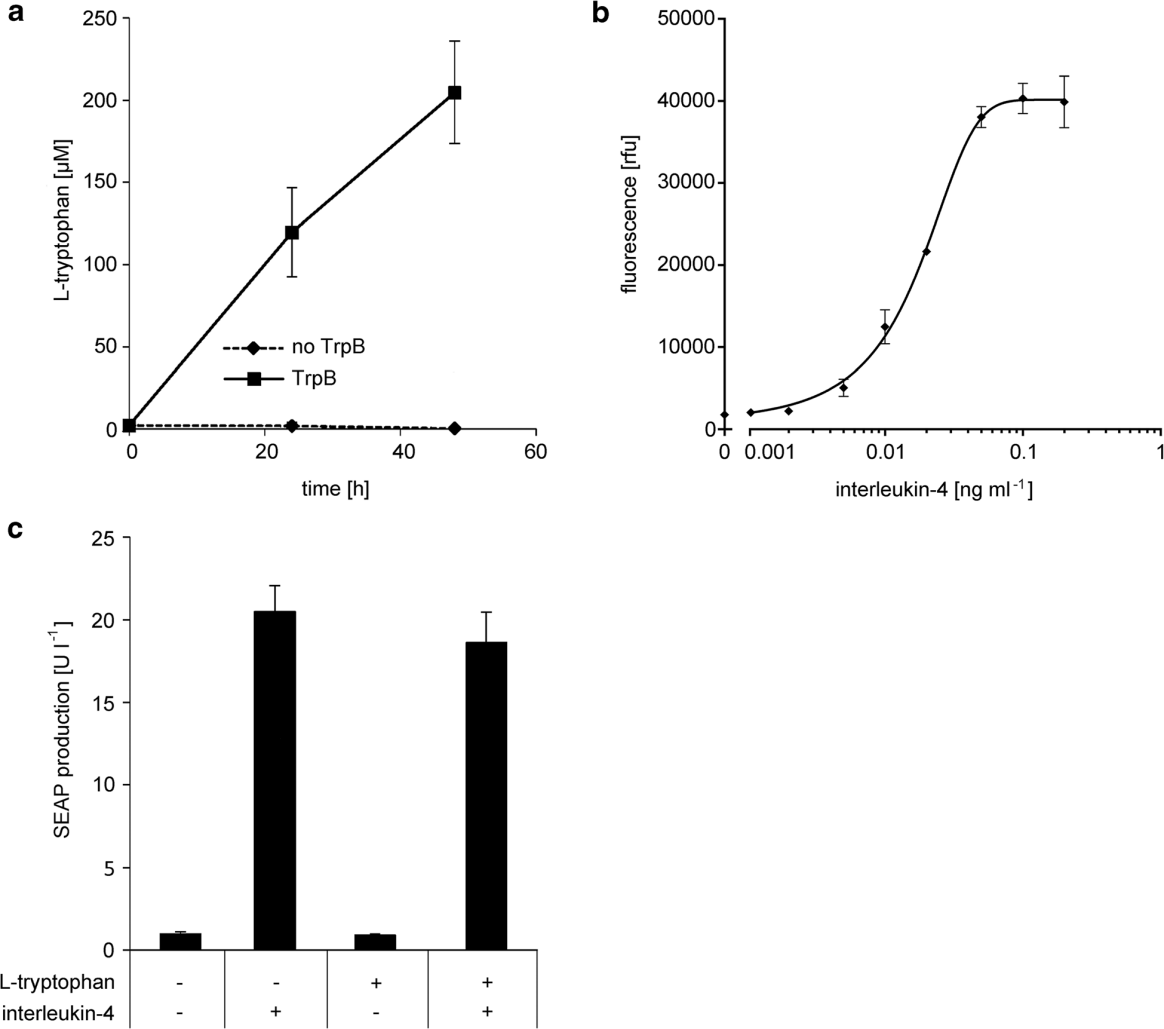
Characterization of the sender/receiver cell population. **a** Quantification of L-tryptophan production. HEK-293T cells with or without constitutively expressed TrpB (pHW074) were cultivated in InVitrus medium supplemented with 500 μM indole. Samples were taken at the indicated points in time and the L-tryptophan production was quantified in the culture medium. **b** Detection of interleukin-4. The sender/receiver cell population (without TrpB, pHW074) was cultivated in the presence of increasing concentrations of interleukin-4 for 48 h prior to quantification of the reporter yellow-fluorescent-protein (YFP). **c** Response to tryptophan and interleukin-4. HEK-293 T cells were transfected for interleukin-4-inducible SEAP production (pSTAT6 and pHW003) and then cultivated in InVitrus medium in the absence or presence of 50 μM L-tryptophan and 1 ng ml^−1^ interleukin-4. After 48 h the production of the SEAP reporter was quantified in the culture medium. Data are means ± SD (*n* = 3)

First, we set out to characterize the sender/receiver cell population with regard to its abilities to produce L-tryptophan and to respond to interleukin-4. To assess the production of L-tryptophan from indole, HEK-293T cells transfected with or lacking the plasmid for constitutive expression of TrpB (pHW074, in a function of the sending module), were cultivated in the presence of 500 μM indole for 48 h. While L-tryptophan-levels of 119.7 μM and 204.7 μM were detected in the cell culture supernatant of TrpB-expressing cells after 24 h and 48 h, respectively, L-tryptophan remained at basal levels in the cultures lacking the TrpB expressing plasmid (Fig. 2a). Next, we analyzed the possibility to tune the response of the sender/receiver cell population via the amount of interleukin-4. This time, HEK-293 T cells were transfected with the plasmids encoding the receiving module (pSTAT6 and pHW040) and cultivated in the presence of increasing concentrations of interleukin-4, which was added to the medium as a purified recombinant protein. Production of the fluorescent reporter protein YFP after 48 h could be increased from the background levels in the absence of interleukin-4 to high fluorescent levels that reached a plateau at approximately 0.1 ng ml^−1^ interleukin-4 (Fig. 2b). To complete the characterization of the sender/receiver cell population, we confirmed that it only responds to the second signal, interleukin-4, but is insensitive to the first signal, L-tryptophan. To this end, we transfected HEK-293T cells for interleukin-4-inducible production of the reporter protein secreted alkaline phosphatase (SEAP) and cultivated the cells in the presence or absence of high concentrations of L-tryptophan or/and interleukin-4. Reporter production was exclusively triggered by interleukin-4, while the presence or absence of L-tryptophan in the culture medium had no effect on the production of the reporter protein (Fig. 2c).

**Fig. 3:**
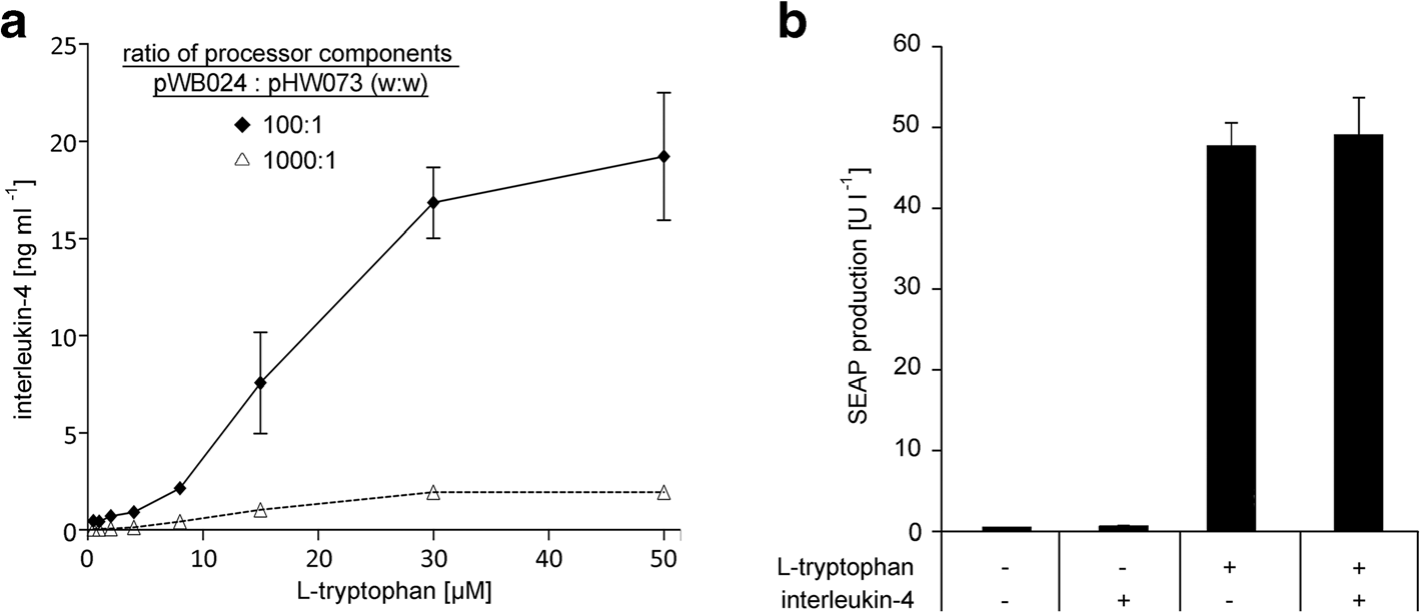
Characterization of the processing cell population. **a** L-tryptophan-induced production of interleukin-4. The processing cell population (pWB024 and pHW073 transfected in ratios (w:w) of 100:1 or 1000:1) was cultivated in InVitrus medium in the presence of increasing concentrations of L-tryptophan. After 48 h interleukin-4 was quantified in the cell culture supernatant. **b** Response to L-tryptophan and interleukin-4. HEK-293T cells were transfected for L-tryptophan-inducible SEAP production (pWB024 and pLMK116) and the cells were cultivated in InVitrus medium in the absence or presence of 50 μM L-tryptophan and 1 ng ml^−1^ interleukin-4. After 48 h the production of the SEAP reporter was quantified in the culture medium. Data are means ± SD (*n* = 3)

Having confirmed the full functionality of the sender/receiver cell population, we moved on to characterize the processing cell population’s ability to respond to L-tryptophan with the production of interleukin-4. To this end, the processing cell population, containing the TrpR-VP16-encoding plasmid (pWB024) in 1000-fold or 100-fold excess (w:w) over the interleukin-4 expression plasmid (pHW073), was cultivated for 48 h in the presence of increasing amounts of L-tryptophan. The quantification of interleukin-4 in the cell culture supernatant revealed rising levels of interleukin-4 in response to increasing concentrations of L-tryptophan for both conditions (Fig. 3a). Theoretically, the width of the edge between the cell populations as visualized by expression of the YFP reporter is directly correlated to the sensitivity of the sender/receiver population towards interleukin-4 and to the production of interleukin-4 by the processing cell line. In particular, the zone of YFP expression will broaden with an increased sensitivity of the sender/receiver population and amount of interleukin-4 produced by the processing population. The analysis of the sender/receiver population had revealed maximal activation of interleukin-4-triggered YFP expression already at interleukin-4 concentrations as low as 0.1 ng ml^−1^ (Fig. 2b). The processing population with the 1000:1 plasmid ratio produced this amount of interleukin-4 in response to L-tryptophan concentrations higher than approximately 5 μM (Fig. 3a, dashed line), whereas the processing cell population with the 100:1 plasmid ratio reached this level already at minute L-tryptophan concentrations (Fig. 3a, solid line). Based on these determinations, combining our sender/receiver cell population with the processing cell population with the 1000:1 plasmid ratio should lead to a sharper YFP expression zone at the population border. However, for the final cell-cell communication network we intended to significantly limit and thereby spatially-define the diffusion of the signaling molecules. This step is crucial for establishment of the system for computation of population borders, but, as a side effect, also substantially reduces the interleukin-4 concentration at the population border. Since the choice of the processing population with the 1000:1 plasmid ratio could thus no longer suffice for the full activation of the sender/receiver cells at the population border (as later confirmed in the supplementary Additional file 1: Figure S1), we decided to proceed with the processing cell population with a 100:1 plasmid ratio. To conclude the characterization of the processing cell population, we confirmed that it only responds to L-tryptophan but not to interleukin-4. To achieve this, we transfected HEK-293T cells for L-tryptophan-responsive SEAP production and cultivated them in the presence of high concentrations of L-tryptophan and/or interleukin-4. After 48 h, SEAP production was only induced by L-tryptophan, while the presence of interleukin-4 had no influence on SEAP levels (Fig. 3b).

**Fig. 4:**
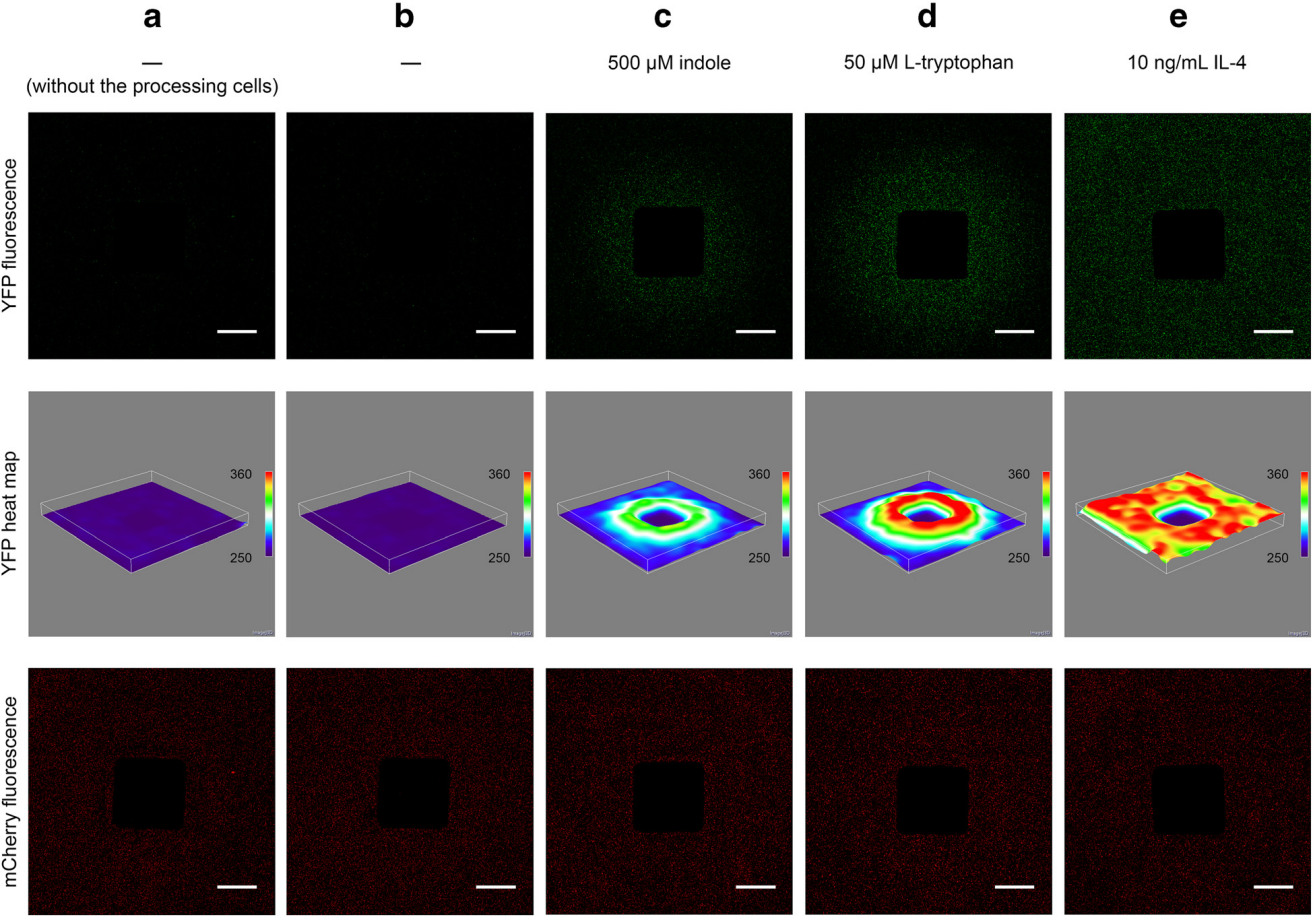
Computation of the border between two mammalian cell populations. The sender/receiver population (pHW074, pSTAT6, pHW040, and pMK047 as a seeding control) was seeded in the outer compartment of a 60 mm culture dish with an insert, while the processing population (pWB024 and pHW073 in a ratio (w:w) of 100:1) was seeded in the inner square-shaped compartment. The cells were overlaid with agar-solidified InVitrus medium supplemented with 500 μM indole and cultivated for 48 h before microscopic analysis of expression of the YFP reporter (column **c**)). Control cells were overlaid with agar-solidified InVitrus medium without indole (column **b**)) or with agar supplemented with 50 μM L-tryptophan (column **d**)) or 10 ng ml^−1^ interleukin-4 (column **e**)). Background level of YFP expression of the sender/receiver cell population is presented in column **a**), where the population was seeded in the absence of processing cell population and medium supplements. The top row shows the expression of the fluorescent reporter protein YFP that is displayed as heat maps in the middle row. The bottom row shows the total cell distribution of the sender/receiver cell population constitutively expressing the fluorescent reporter protein mCherry, whereas the dark squares in the center are the inner compartments. Scale bar = 5 mm

After characterization and optimization of the sender/receiver cell population as well of the processing cell population we set up the synthetic cell-cell communication network for the detection of population borders. First, the sender/receiver and the processing cell population were seeded in separate compartments of a cell culture dish. Following attachment of the cells, the mechanical border between the populations was removed and the cells were overlaid with agarose-solidified InVitrus medium supplemented with 500 μM indole, to limit diffusion of the signaling molecules. After 48 h, YFP expression was clearly restricted to the border between the two cell populations (Fig. 4c), while the control cells that had been cultured in the presence of 50 μM L-tryptophan (Fig. 4d) or of 10 ng ml^−1^ interleukin-4 (Fig. 4e) showed wider range or full area expression of YFP, respectively. In comparison to the Fig. 4c, a substantially more defined but also dimmer YFP detection of the population border was observed when the processing cell population with a 100:1 plasmid ratio was replaced with the one of a 1000:1 (Additional file 1: Figure S1). On the other hand, the control cells that received no supplements (Fig. 4b), showed no significant expression of YFP, even in comparison to the background fluorescence of the sender/receiver population, which was seeded in the absence of both the processing cell population and any supplements in Fig. 4a.

Beyond this proof-of-principle, our synthetic network opens up the possibility to emulate intercellular communication systems that require the computation of population borders as sharper or wider edges, simply by tuning key parameters of the system. The high flexibility of our system allows the visualized edge to be decreased in broadness by 1) decreasing the production of L-tryptophan in the sender/receiver population, 2) decreasing the sensitivity of the processing cell population towards L-tryptophan, 3) decreasing the production of interleukin-4 by the processing cell population, 4) decreasing the sensitivity of the sender/receiver population towards interleukin-4 and by 5) limiting the diffusion of interleukin-4. Analogously, the spread of the detected edge may be increased by adjusting the parameters in the opposite direction.

## Conclusion

We have established the first synthetic mammalian system for two-way cell-cell communication to compute and visualize the border between cell populations. Communication between the populations takes place via L-tryptophan and interleukin-4 and results in the production of a fluorescent protein at the population border. Formation and maintenance of borders between functionally different mammalian cell populations are highly interesting and complex phenomena, which are especially vital in the context of embryonic development and homeostasis [15, 16]. It can be expected that this network will be useful as a tool and a blueprint to help emulate these processes in order to increase our understanding of the molecular mechanisms that underlie tissue formation, as well as contribute to the engineering of artificial tissues.

## Methods

### DNA cloning

The construction of expression vectors is given in detail in Additional file 2: Table S1.

### Cell culture and transfection

All experiments were conducted in human embryonic kidney fibroblasts (HEK-293T [17]). Unless indicated, the cells were maintained in Dulbecco’s modified Eagle’s medium (PAN, cat. no. P03-0710) supplemented with 10 % (v/v) FBS (PAN, cat. no. P30-3602, batch no. P101003TC), 100 U ml^−1^ of penicillin and 0.1 mg ml^−1^ of streptomycin (PAN, cat no. P06-07001). In L-tryptophan-sensitive experiments the cells were cultured in L-tryptophan free InVitrus medium (Cell Culture Technologies, custom-made) supplemented with 10 % (v/v) FBS, 100 U ml^−1^ of penicillin, 0.1 mg ml^−1^ of streptomycin and 0.5 μM L-tryptophan. Where indicated, L-tryptophan or indole was added to the culture medium from a 39.17 mM stock in H_2_O or from a 500 mM stock in ethanol, respectively.

Cells were transfected using an optimized polyethylene-imine-based method (PEI, linear, MW: 25 kDa) (Polyscience) [18]. In brief, 1 mg ml^−1^ PEI solution in H_2_O was adjusted to pH 7.0 with HCl, sterile filtered and stored at −80 °C in aliquots. Next, 70,000 cells were seeded per well of a 24-well plate and cultivated overnight. Aliquots of 0.75 μg of DNA were diluted in 50 μl of OptiMEM (Invitrogen) and mixed with 2.5 μl of PEI solution in 50 μl of OptiMEM by vortexing (amounts scaled to one well). After 20 min incubation at room temperature, the precipitate was added to the cells. The culture medium was replaced 5 h after the transfection. Unless indicated, plasmids were transfected in equal amounts (w:w).

### Production of fibronectin

His-tagged fibronectin (domains 7–10) was expressed from pET15bFN-III_7-10_RGE [19] in *E.coli BL21(DE3)pLysS* (Promega, cat. no. L1195) and purified using Ni-NTA chromatography. Aliquots of the purified protein were frozen in liquid nitrogen and stored in PBS (pH 7.4) at a concentration of 1 mg ml^−1^ at −80 °C.

### Computation of the interface between adjacent cell populations

To set up a culture system with two compartments, the inner wall of a culture insert (Ibidi, cat. no. 80209) was removed and the modified insert was placed in the middle of a 60 mm cell culture dish. Next, the surface of the culture dish was coated with fibronectin by applying 37.5 μl of fibronectin solution (80 μg ml^−1^) to the central area and 1.5 ml to the outer area. After incubation for 1 h at room temperature the processing cell population (HEK-293T transfected with pWB024 and pHW073, 24 h post transfection) was seeded in the inner compartment (70,000 cells in 125 μl InVitrus medium), while the sender/receiver population (HEK-293T transfected with pHW074, pHW040, pSTAT6 and pMK47 – as a transfection control plasmid for constitutive expression of mCherry; 1.5 μg per 10 cm culture dish transfection – 24 h post transfection) was seeded in the outer compartment (2.75 million cells in 5 ml InVitrus medium). Twenty-four hours later, the inserts were removed and the cells were overlaid with 5.25 ml agarose-solidified InVitrus medium (1 % w/v) supplemented with 500 μM indole. After 35 min at room temperature for solidification of the overlay, the cells were cultivated at 37 °C for 48 h before microscopic detection of the fluorescent reporter proteins.

### Reporter assays

The reporter SEAP was quantified in the cell culture medium, using a colorimetric assay as described elsewhere [18]. Interleukin-4 (IL-4) and L-tryptophan were quantified in the culture medium using an IL-4 ELISA kit (Pepro Tech, cat. no. 900-K14) or the Bridge-IT L-Tryptophan Fluorescence Assay (Mediomics, cat. no. 1-1-1002A) according to the manufacturer’s instructions, respectively.

The fluorescence intensity of mammalian cells was quantified in cell lysates. First, the cells were lysed by the addition of 250 μl lysis buffer (25 mM Tris–HCl pH 7.8, 1 % (v/v) Triton X-100, 15 mM MgSO_4_, 4 mM EGTA, 1 mM DTT) per well of a 24-well plate. Then, 100 μl of each lysate was transferred to a 96-well flat-bottom black plate and YFP fluorescence intensity was quantified using a Synergy 4 multimode microplate reader (BioTek Instruments) or a Tecan infinite 200Pro microplate reader (Tecan Group), with excitation at 490 nm and emission at 527 nm.

A fluorescence microscope (Zeiss Cell Observer, Carl Zeiss) was used to acquire YFP and mCherry mosaic images of 60 mm dishes with a Plan-Neofluar pol. 2.5× objective lens (NA 0.075). The same exposure time was used for all samples. Mosaic images were stitched with XuvStitch (v1.8.1-beta5) and processed with Fiji (ImageJ v2.0.0-rc-41/1.50b), where heat maps were produced using the 3D Surface Plot function.

## Availability of supporting data

The data sets supporting the results of this article are included within the article (and its additional files).

## Additional files

Additional file 1: Figure S1. Tunability of the edge visualization between two mammalian cell populations. The experiment was performed in the same way as the one in Fig. 4c, only that this time the processing cell population, seeded in the inner compartment, contained the plasmids pWB024 and pHW073 in a ratio of 1000:1 (w:w). Scale bar = 5 mm. (TIF 5327 kb) Additional file 2: Table S1. Expression vectors and oligonucleotides designed and used in this study. (DOCX 35 kb)
