# Supplementary figures and images for: A synthetic mammalian network to compute population borders based on engineered reciprocal cell-cell communication
Source: BMC Syst Biol. 2015 Dec 30;9:97. doi: 10.1186/s12918-015-0252-1 (PMC4696150; doi:10.1186/s12918-015-0252-1)

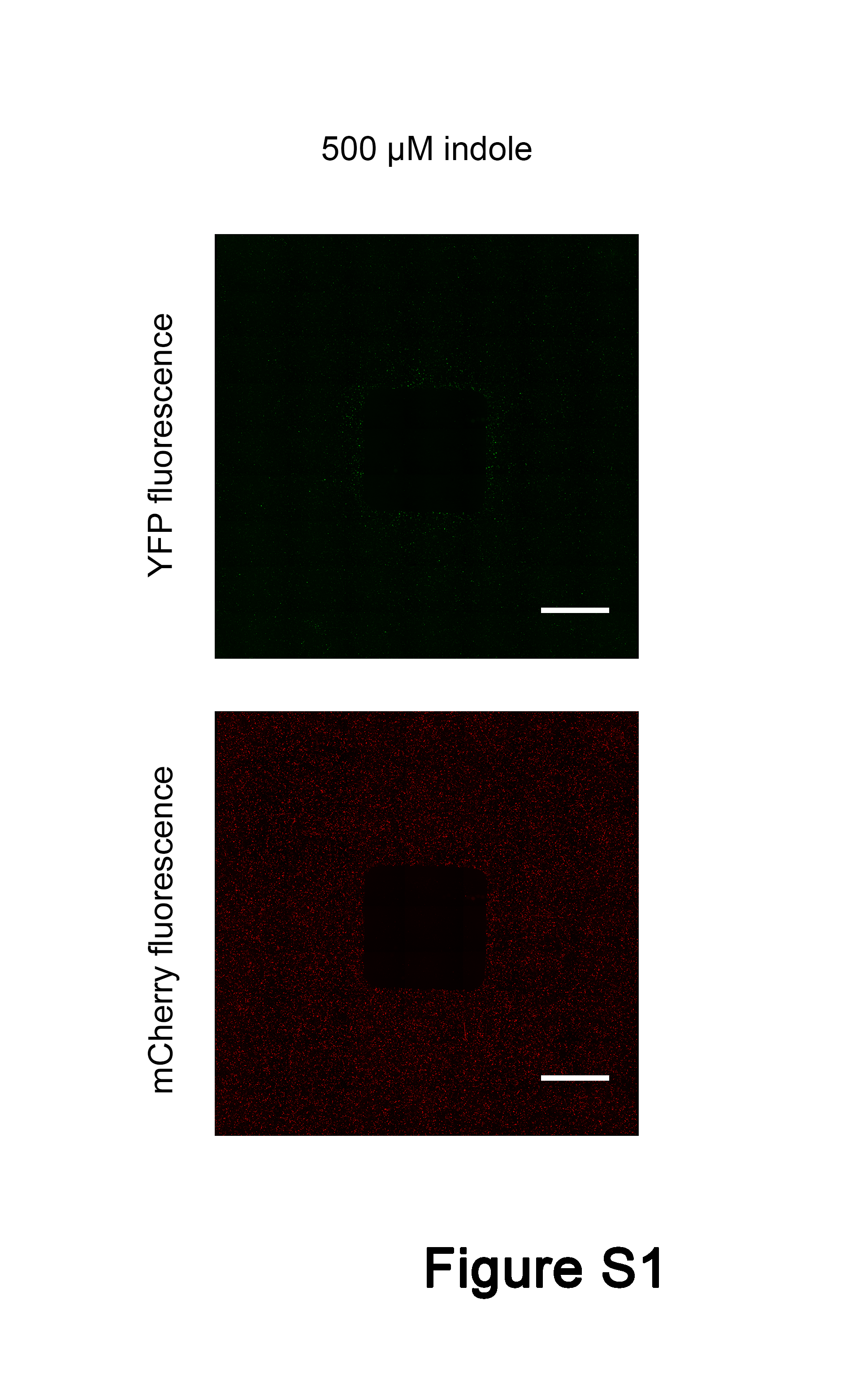

Supplement: Additional file 1: Figure S1. — Tunability of the edge visualization between two mammalian cell populations. The experiment was performed in the same way as the one in Fig. 4c, only that this time the processing cell population, seeded in the inner compartment, contained the plasmids pWB024 and pHW073 in a ratio of 1000:1 (w:w). Scale bar = 5 mm. (TIF 5327 kb) [file 12918_2015_252_MOESM1_ESM.tif]
