# Supplementary material for: A synthetic mammalian network to compute population borders based on engineered reciprocal cell-cell communication
Source: BMC Syst Biol. 2015 Dec 30;9:97. doi: 10.1186/s12918-015-0252-1 (PMC4696150; doi:10.1186/s12918-015-0252-1)
Supplement: Additional file 2: Table S1. — Expression vectors and oligonucleotides designed and used in this study. (DOCX 35 kb) [file 12918_2015_252_MOESM2_ESM.docx]

**A synthetic mammalian network to compute population borders based on engineered reciprocal cell-cell communication**

Katja Kolar^1^, Hanna M. Wischhusen^1,2^, Konrad Müller^1,3^, Maria Karlsson^1,4^, Wilfried Weber^1,5^ and Matias D. Zurbriggen^1,6^*

^1^Faculty of Biology, University of Freiburg, DE-79104 Freiburg, Germany

^2^Current address: DMK GmbH, Head of Quality Assurance, DE-26939 Ovelgönne, Germany

^3^Current address: Novartis Pharma AG, Biologics Process R&D, CH-4002 Basel, Switzerland

^4^Current address: Respiratory, Inflammation and Autoimmunity (RIA) iMED, AstraZeneca, SE-431 83 Mölndal, Sweden

^5^BIOSS Centre for Biological Signalling Studies, University of Freiburg, DE-79104 Freiburg, Germany

^6^Current address: Institute of Synthetic Biology and Cluster of Excellence on Plant Science (CEPLAS), University of Düsseldorf, DE-40225 Düsseldorf, Germany

* Corresponding author: Tel: +49 211 81 15015; E-mail: matias.zurbriggen@uni-duesseldorf.de

**Table S1** Expression vectors and oligonucleotides designed and used in this study.

| Plasmid | Description | Reference or source |
| --- | --- | --- |
| pHW003 | P_STAT6_-*seap*-pA | [1] |
| pHW040 | P_STAT6_-*yfp*-pA | [2] |
| pLMK109 | _TRP_O_2_-P_hCMVmin_-*trpβ*-pA (_TRP_O_2_-P_hCMVmin_, P_TRP_)  P_hCMVmin_ was amplified from pMF111[3] using oligos oLMK115 (5’‑ccggtcgtcgacag*attgtaatattatagcattacaattgtaatattatagcattacaa*ggatcctgcagGTCGAGCTCGGTACCCGGGT‑3’) and oLMK116 (5’‑aataggggttaagtaatgttgtcatggtgCGAATTCGGGGCCGCGGAGG‑3’), while *trpβ* was amplified from E. coli K-12 MG1655 genomic DNA using oligos oLMK117 (5’‑gcaccATGACAACATTACTTAACCCCTATTTTGGTGA‑3’) and oLMK118 (5’‑tacagggcggccgcTCAGATTTCCCCTCGTGCTTTCAAAATATCG‑3’). Both products were fused using oligos oLMK119 (5’‑ccggtcgtcgacAGATTGTAATATTATAG‑3’) and oLMK118, digested (*Sal*I/*Not*I) and ligated into (*Sal*I/*Not*I) pWW1088[4]. | This work |
| pLMK116 | P_TRP_-*seap*-pA  SEAP was excised from pWW192[5] (*Eco*RI/*Not*I) and ligated (*Eco*RI/*Not*I) into pLMK109. | This work |
| pHW073 | P_TRP_-*il‑4*-pA  Il-4 was amplified from cDNA (Open Biosystems, cat no: MHS1010-97228538) using oligos oHW004 (5’‑tcatgcggccgcTCAGCTCGAACACTTTGAATA‑3’) and oHW136 (5’‑caccatccgcggcaccATGGGTCTCACCTCCCAAC‑3’), digested (*Sac*II/*Not*I) and ligated (*Sac*II/*Not*I) into pLMK109. | This work |
| pHW074 | P_EF1α_-*trpß*-pA  Trpβ was excised (*Eco*RI/*Not*I) from pLMK109 and ligated (*Eco*RI/*Not*I) into pWW029[6]. | This work |
| pSTAT6 | P_hCMV_-*stat6*-pA  Open Biosystems cat. no.: MHS1010-97228301. | Open Biosystems |
| pWB024 | P_SV40_-*trpR-vp16*-pA | [7] |
| pET15bFN-III_7-10_RGE | P_T7_-*his_6_-fn-III_7-10_RGE*-pA | [8] |
| pMK047 | P_EF1_-*mCherry*-pA | [9] |

E, macrolide-responsive repressor protein; FN, fibronectin; Il‑4, human interleukin‑4; pA, polyadenylation signal; P_AIR_, acetaldehyde–responsive promoter; P_BIT1_, biotin-responsive promoter;P_EF1α_, human elongation factor 1α promoter; P_hCMV_, human cytomegalovirus immediate early promoter; P_hCMVmin_, minimal human cytomegalovirus immediate early promoter; P_STAT6_, human eotaxin-3 promoter; P_SV40_, Simian virus 40 early promoter; P_TRP_, tryptophan-responsive promoter; SEAP, human secreted alkaline phosphatase; STAT6, signal transducer and activator of transcription 6; TrpB, *E. coli* tryptophan synthase subunit ß; TrpR, tryptophan repressor protein; _TrpR_O_2_, operator sequence binding TrpR; VP16, *Herpes simplex* virus-derived transactivation domain; YFP, yellow fluorescent protein; mCherry, red monomeric fluorescent protein.

Uppercase sequence in oligos, annealing sequence; underlined sequence, restriction site; italic sequence, _TrpR_O_2_ operator sequence.

1. Christen EH, Karlsson M, Kämpf MM, Schoenmakers R, Gübeli RJ, Wischhusen HM, Friedrich C, Fussenegger M, Weber W: **Conditional DNA-protein interactions confer stimulus-sensing properties to biohybrid materials**. *Adv Funct Mater* 2011, **21**:2861–2867.

2. Lienemann PS, Karlsson M, Sala A, Wischhusen HM, Weber FE, Zimmermann R, Weber W, Lutolf MP, Ehrbar M: **A Versatile Approach to Engineering Biomolecule-Presenting Cellular Microenvironments**. *Adv Healthc Mater* 2013, **2**:292–296.

3. Fussenegger M, Mazur X, Bailey JE: **A novel cytostatic process enhances the productivity of Chinese hamster ovary cells**. *Biotechnol Bioeng* 1997, **55**:927–939.

4. Weber W, Lienhart C, Daoud-El Baba M, Fussenegger M: **A biotin-triggered genetic switch in mammalian cells and mice**. *Metab Eng* 2009, **11**:117–124.

5. Weber W, Rimann M, Spielmann M, Keller B, Baba MD, Aubel D, Weber CC, Fussenegger M: **Gas-inducible transgene expression in mammalian cells and mice**. 2004, **22**:1440–1444.

6. Weber W, Fux C, Daoud-el Baba M, Keller B, Weber CC, Kramer BP, Heinzen C, Aubel D, Bailey JE, Fussenegger M: **Macrolide-based transgene control in mammalian cells and mice.** *Nat Biotechnol* 2002, **20**:901–907.

7. Bacchus W, Lang M, El-Baba MD, Weber W, Stelling J, Fussenegger M: **Synthetic two-way communication between mammalian cells**. *Nat Biotechnol* 2012, **30**:991–996.

8. Takahashi S, Leiss M, Moser M, Ohashi T, Kitao T, Heckmann D, Pfeifer A, Kessler H, Takagi J, Erickson HP, Fässler R: **The RGD motif in fibronectin is essential for development but dispensable for fibril assembly**. *J Cell Biol* 2007, **178**:167–178.

9. Müller K, Engesser R, Metzger S, Schulz S, Kämpf MM, Busacker M, Steinberg T, Tomakidi P, Ehrbar M, Nagy F, Timmer J, Zubriggen MD, Weber W: **A red/far-red light-responsive bi-stable toggle switch to control gene expression in mammalian cells.** *Nucleic Acids Res* 2013, **41**:e77.
